# Supplementary material for: Larval shell chemistry of the Olympia oyster (Ostrea lurida) in Puget Sound, WA to assess population connectivity and restoration planning
Source: PLoS One. 2025 Apr 8;20(4):e0320136. doi: 10.1371/journal.pone.0320136 (PMC11977958; doi:10.1371/journal.pone.0320136)
Supplement: S1 Appendix — (DOCX) [file pone.0320136.s001.docx]

# S1 Appendix. Supplemental materials including methods, figures, and tables.

# Methods

## Assignment of settler signatures to brood origins

To assess whether brood and settler signatures were comparable, we estimated the proportion of settlers unlikely to have originated from brood sources following the methods used in Standish et al. 2008 [1].  Using the combined Mn:Ca and Sr:Ca elemental signature, a bivariate cumulative probability density was estimated for each settler and potential source based on the means and covariances for each brood region.  Mn:Ca and Sr:Ca were used in this analysis because they provided the most discriminatory power among brood regions in the LDA analysis.  Settlers were deemed as likely to have originated from outside of the characterized brood sources, or to have signatures distinct from the sampled brooded larvae if they had probabilities <5% for all sampled brood regions.

**Settler signature cluster analysis**

We used a Markov-chain Monte Carlo (MCMC) clustering algorithm [2] to identify the number of distinct clusters of elemental signatures (brood sources) present in the *O. lurida* settler cohort, estimate the contribution of each putative brood sources to the settler populations, and assign individual settlers to brood sources.  The MCMC clustering algorithm simultaneously estimates brood source parameters (means and covariances of elemental signatures from each putative brood source, or cluster), mixture probabilities (the proportion of the settlers drawn from each putative brood source), and generates brood source assignment probabilities for each individual.  The MCMC procedure is repeated across a range of cluster numbers, and post-hoc model selection is conducted using a variation of the Deviance Information Criterion (DIC3) [3, 4], which is the Bayesian equivalent of the Akaike Information Criterion (AIC).

The MCMC clustering algorithm was conducted using MATLAB 9.2 (Mathworks, Natick, MA) according to the procedures outlined in [2] and [5].  Settler elemental signatures for MCMC analysis consisted of the 3 elements with the most discriminatory power among brood regions as identified in the LDA analysis (Mn:Ca, Sr:Ca, Fe:Ca), and were analyzed with both settler collection regions combined (Fidalgo Bay and Dyes Inlet).  This reduced combination of variables was applied to avoid model overparameterization when analyzing the complete sample of settlers.  Settler elemental signatures consisting of the full suite of 8 elements found to produce the best brood region discrimination with LDA (Al:Ca, P:Ca, Mn:Ca, Fe:Ca, Zn:Ca, Sr:Ca, Ba:Ca, U:Ca) were subsequently analyzed separately for Fidalgo Bay and Dyes Inlet region settlers.  MCMC results for the 8-element signatures produced similar results and are not presented here.  Each MCMC run was conducted using uninformative priors, with 150,000 iterations per run, discarding the first 50,000 iterations as burn-in, and followed by 50,000 iterations of a post-hoc relabeling algorithm.  We evaluated MCMC runs with the number of clusters (*K*), representing the number of putative brood sources, ranging from 1 to 4.  Multiple replicates of MCMC runs were conducted for each value of *K* to verify convergence.

# Supplementary Figures

**S1_Figure.tif.** Elemental ratios to calcium for the 8 elements used to classify brooded larval shells of O. lurida with linear discriminant analysis (LDA) from the three regions North Sound (NS), Sequim Bay (Sequim) and Central Sound (CS) in Puget Sound, WA. Horizontal lines represent median values; lower and upper hinges represent the 25th and 75th percentiles, respectively; whiskers extend to the largest and smallest measured value within 1.5 x interquartile range (IQR; difference between 75th and 25th percentile); filled circles represent outliers beyond 1.5 x IQR (some upper limits truncated for ease of visualization). The results of individual ANOVAs (df=2) are shown as p value and different letters above bars indicate significant differences (p < 0.05) from Tukey post hoc tests. Elemental ratios of settled recruits (Set) of unknown brood origin are plotted here (unshaded boxes) for comparison but were not included in the ANOVA.

**S2_Figure.tif.** NMDS ordination of elemental concentrations in brooded larval shells collected using non-lethal MgSO_4_ anesthetic and lethal collection. Left plot analysis are labeled to sample collection method and the right plot analysis are labeled according to the Olympia oyster the larvae were collected from.

# Supplementary Tables

Table A. Summary of late stage brooded *O. lurida* larval samples collected for elemental fingerprinting.

|  |  | early | | | | | | | late | | | | | | | | | | |
| --- | --- | --- | --- | --- | --- | --- | --- | --- | --- | --- | --- | --- | --- | --- | --- | --- | --- | --- | --- |
| Region | Oyster Collection Site | 6/15 | 6/17 | 6/19 | 6/29 | 6/30 | 7/1 | 7/2 | 7/6 | 7/7 | 7/9 | 7/14 | 7/16 | 7/17 | 7/22 | 7/28 | 7/29 | 7/30 | 8/4 |
| **North Sound** n = 33 | **Fidalgo Bay** |  | 1 |  |  | 3 | 5 |  |  | 3 |  |  |  |  |  |  | 5 |  | 1 |
|  | Similk Bay |  |  |  |  |  |  |  |  | 4 |  | 2 |  |  | 1 | 1 |  |  | 4 |
|  | Samish Bay |  |  |  |  |  |  |  |  |  |  |  | 1 |  |  |  | 2 |  |  |
| **Central Sound** n = 49 | **Dyes Inlet** |  |  | 2 |  |  |  | 13 |  |  | 1 |  | 6 |  |  |  |  | 7 |  |
|  | Port Gamble | 3 |  |  |  |  |  |  | 3 |  |  |  |  |  |  |  |  |  |  |
|  | Liberty Bay |  |  |  |  |  |  |  |  |  |  |  | 4 | 2 |  |  |  | 2 |  |
| **Sequim** n = 12 | Sequim Bay |  |  |  |  |  | 12 |  |  |  |  |  |  |  |  |  |  |  |  |
| **South Sound** | Little Skookum Inlet |  |  |  | 5 |  |  |  |  |  |  |  |  |  |  |  |  |  |  |

Table B.  External precisions (%) for trace elements based on relative standard deviations of the reference material (MACS-3). Limits of detection (LOD, umol mol-1) were calculated as the mean detection limit across all *O. lurida* brood shell ablations for each element, as reported by Glitter software.

| Element:Ca | Precision | LOD |
| --- | --- | --- |
| Mg:Ca | 7.6 | 46.68 |
| Al:Ca | 10.1 | 5.25 |
| P:Ca | 18 | 115.49 |
| Mn:Ca | 4.1 | 2.68 |
| Fe:Ca | 7.9 | 8.33 |
| Cu:Ca | 6.5 | 4.90 |
| Zn:Ca | 11.8 | 26.39 |
| Ga:Ca | 8.3 | 0.26 |
| Sr:Ca | 8.7 | 0.32 |
| Ba:Ca | 6.2 | 0.38 |
| Pb:Ca | 7.5 | 0.09 |
| U:Ca | 11.8 | 0.02 |

Table C. Canonical coefficients for linear discriminant analysis conducted on *O. lurida* brood shell elemental signatures among regions, representing the relative discriminatory power of each elemental variable.

| **Element** | **LD1** | **LD2** |
| --- | --- | --- |
| Sr:Ca | 0.535 | -0.648 |
| Fe:Ca | 0.484 | -0.743 |
| P:Ca | 0.478 | -0.557 |
| Al:Ca | 0.283 | 0.085 |
| U:Ca | -0.171 | 0.992 |
| Ba:Ca | -0.442 | -0.29 |
| Zn:Ca | -0.615 | 0.012 |
| Mn:Ca | -0.706 | -0.341 |

Table D. Summary of model selection for MCMC analysis of the number of brood sources contributing to *O. lurida* datasets, as estimated with settler natal shell elemental signatures (Mn:Ca, Sr:Ca, Fe:Ca). Settler signatures (unknown brood origins) from Fidalgo Bay and Dyes Inlet were analyzed together. Clusters (K) representing brood sources ranged from 1 to 4. DIC3 (mean ± SD for n replicates) was used to select the best-fit model, represented in bold. The proportion of individuals assigned to each brood cluster (1 - 4) is calculated as a percentage (mean ± SD) for clusters ranked by occupancy among model runs.

|  |  |  | Proportion of individuals assigned to brood cluster (%) | | | |
| --- | --- | --- | --- | --- | --- | --- |
| *K* | DIC3 | n | 1 | 2 | 3 | 4 |
|  |  |  |  |  |  |  |
| **1** | **1245.7 ± 0** | **5** | **100 ± 0.0** | - | - | - |
| 2 | 1622.5 ± 31.5 | 5 | 93.6 ± 14.2 | 6.4 ± 14.2 | - | - |
| 3 | 1871.5 ± 28.4 | 5 | 64.3 ± 9.4 | 35.5 ± 9.6 | 0.3 ± 0.6 | - |
| 4 | 2127.3 ± 25.2 | 5 | 88.6 ± 5.3 | 10.9 ± 4.9 | 0.5 ± 0.7 | 0 ± 0 |
|  |  |  |  |  |  |  |

## References

1. Standish JD, Sheehy M, Warner RR. Use of otolith natal elemental signatures as natural tags to evaluate connectivity among open-coast fish populations. Mar Ecol Prog Ser. 2008;356:259-68.
2. White JW, Standish JD, Thorrold SR, Warner RR. Markov Chain Monte Carlo methods for assigning larvae to natal sites using natural geochemical tags. Ecol Appl. 2008;18(8):1901-13.
3. Celeux G, Forbes F, Robert CP, Titterington DM. Deviance information criteria for missing data models. Bayesian Anal. 2006;1(4):651-73.
4. Spiegelhalter DJ, Best NG, Carlin BP, Van Der Linde A. Bayesian measures of model complexity and fit. J R Stat Soc Series B Stat Methodol. 2002;64(4):583-639.
5. Barnett BK, Patterson WF, Kellison T, Garner SB, Shiller AM. Potential sources of red snapper *Lutjanus campechanus* recruits estimated with Markov Chain Monte Carlo analysis of otolith chemical signatures. Mar Freshw Res. 2016;67(7):992-1001.
