# Supplementary figures and images for: Larval shell chemistry of the Olympia oyster (Ostrea lurida) in Puget Sound, WA to assess population connectivity and restoration planning
Source: PLoS One. 2025 Apr 8;20(4):e0320136. doi: 10.1371/journal.pone.0320136 (PMC11977958; doi:10.1371/journal.pone.0320136)

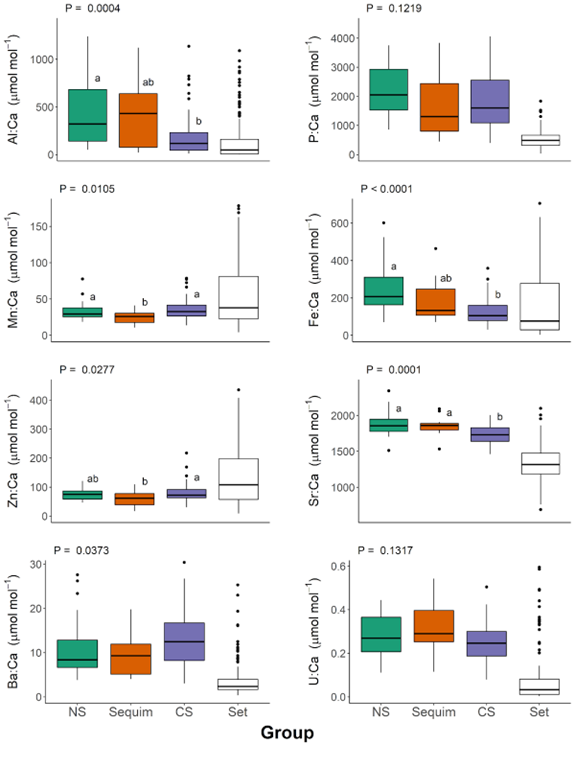

Supplement: S1 Fig — Horizontal lines represent median values; lower and upper hinges represent the 25th and 75th percentiles, respectively; whiskers extend to the largest and smallest measured value within 1.5 x interquartile range (IQR; difference between 75th and 25th percentile); filled circles represent outliers beyond 1.5 x IQR (some upper limits truncated for ease of visualization). The results of individual ANOVAs (df = 2) are shown as p value and different letters above bars indicate significant differences (p < 0.05) from Tukey post hoc tests. Elemental ratios of settled recruits (Set) of unknown brood origin are plotted here (unshaded boxes) for comparison but were not included in the ANOVA. (TIF) [file pone.0320136.s003.tif]

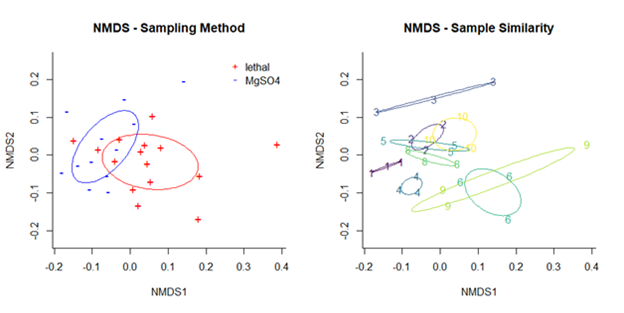

Supplement: S2 Fig — Left plot analysis are labeled to sample collection method and the right plot analysis are labeled according to the Olympia oyster the larvae were collected from. (TIF) [file pone.0320136.s004.tif]
